# A pharmacovigilance study on antibody-drug conjugate (ADC)-related neurotoxicity based on the FDA adverse event reporting system (FAERS)

**DOI:** 10.3389/fphar.2024.1362484

**Published:** 2024-02-07

**Authors:** Linlin Tang, Cuicui Sun, Wenshan Liu, Haiyan Wu, Chuanhua Ding

**Affiliations:** ^1^ Department of Pharmacy, Affiliated Hospital of Weifang Medical University, Weifang, China; ^2^ Department of Pharmacy, Qilu Hospital of Shandong University, Ji’nan, China; ^3^ Department of Pharmacy, Central Hospital Affiliated to Shandong First Medical University, Ji’nan, China

**Keywords:** antibody-drug conjugates (ADCs), neurotoxicity, FAERS, pharmacovigilance, data mining

## Abstract

**Background:** Antibody-drug conjugates (ADCs) are a relatively new class of anticancer agents that use monoclonal antibodies to specifically recognize tumour cell surface antigens. However, off-target effects may lead to severe adverse events. This study evaluated the neurotoxicity of ADCs using the FDA Adverse Event Reporting System (FAERS) database.

**Research design and methods:** Data were extracted from the FAERS database for 2004 Q1 to 2022 Q4. We analysed the clinical characteristics of ADC-related neurological adverse events (AEs). We used the reporting odds ratio (ROR) and proportional reporting ratio (PRR) for the disproportionality analysis to evaluate the potential association between AEs and ADCs.

**Results:** A total of 562 cases of neurological AEs were attributed to ADCs. The median age was 65 years old [(Min; Max) = 3; 92]. Neurotoxic signals were detected in patients receiving brentuximab vedotin, enfortumab vedotin, polatuzumab vedotin, trastuzumab emtansine, gemtuzumab ozogamicin, inotuzumab ozogamicin, and trastuzumab deruxtecan. The payloads of brentuximab vedotin, enfortumab vedotin, polatuzumab vedotin, and trastuzumab emtansine were microtubule polymerization inhibitors, which are more likely to develop neurotoxicity. We also found that brentuximab vedotin- and gemtuzumab ozogamicin-related neurological AEs were more likely to result in serious outcomes. The eight most common ADC-related nervous system AE signals were peripheral neuropathy [ROR (95% CI) = 16.98 (14.94–19.30), PRR (95% CI) = 16.0 (14.21–18.09)], cerebral haemorrhage [ROR (95% CI) = 9.45 (7.01–12.73), PRR (95% CI) = 9.32 (6.95–12.50)], peripheral sensory neuropathy [ROR (95% CI) = 47.87 (33.13–69.19), PRR (95% CI) = 47.43 (32.93–68.30)], polyneuropathy [ROR (95% CI) = 26.01 (18.61–36.33), PRR (95% CI) = 25.75 (18.50–35.86)], encephalopathy [ROR (95% CI) = 5.16 (3.32–8.01), PRR (95% CI) = 5.14 (3.32–7.96)], progressive multifocal leukoencephalopathy [ROR (95% CI) = 22.67 (14.05–36.58), PRR (95% CI) = 22.52 (14.01–36.21)], taste disorder [ROR (95% CI) = 26.09 (15.92–42.76), PRR (95% CI) = 25.78 (15.83–42.00)], and guillain barrier syndrome [ROR (95% CI) = 17.844 (10.11–31.51), PRR (95% CI) = 17.79 (10.09–31.35)]. The mortality rate appeared to be relatively high concomitantly with AEs in the central nervous system.

**Conclusion:** ADCs may increase the risk of neurotoxicity in cancer patients, leading to serious mortality. With the widespread application of newly launched ADC drugs, combining the FAERS data with other data sources is crucial for monitoring the neurotoxicity of ADCs. Further studies on the potential mechanisms and preventive measures for ADC-related neurotoxicity are necessary.

## 1 Introduction

Antibody-drug conjugates (ADCs) are a class of drugs composed of monoclonal antibodies, linkers, and cytotoxic drugs (payloads). Monoclonal antibodies recognize cancer cell surface antigens and deliver highly effective cytotoxic drugs specifically to the tumour cells, thereby achieving efficient therapeutic effects and low toxicity (Tarantino et al., 2022).

The Food and Drug Administration (FDA) has approved 12 types of ADCs for the treatment of haematological and solid tumours. Gemtuzumab ozogamicin (GO) is indicated for CD33-positive acute myeloid leukemia (AML). The FDA authorized it in 2000, delisted it in 2010, and then gave it another approval in 2017. It was approved by the European Medicines Agency (EMA) in 2018. In 2017, the FDA and EMA authorized inotuzumab ozogamicin (IO) for the treatment of refractory B-cell precursor acute lymphoblastic leukemia (ALL) (Sun et al., 2023). Trastuzumab deruxtecan (TD) was approved by the FDA in 2019 and the EMA in 2021. Its indications include metastatic ErbB2 positive breast cancer, non-small cell lung cancer (NSCLC), and gastric or gastroesophageal junction adenocarcinoma (FDALabel, 2022c; Van Cutsem et al., 2023). Triple negative breast cancer is the approved indication for sacituzumab govitecan (SG), which received FDA and EMA approval in 2020 and 2021, respectively. Triple negative breast cancer is the approved indication for loncastuximab tesirine (LT), which received FDA and EMA approval in 2021 and 2022, respectively. The indications for trastuzumab emtansine (TE), enfortumab vedotin (EV), brentuximab vedotin (BV), and polatuzumab vedotin (PV) are ErbB2-positive metabolic breast cancer, advanced urothelial cancer, Hodgkin’s lymphoma/analytical large cell lymphoma/peripheral T-cell lymphomas/mycosis fungoides, or diffuse large B-cell lymphoma. The TE, EV, BV, and PV were approved by FDA in 2013, 2019, 2011, or 2019 and EMA in 2013, 2022, 2012, or 2020, respectively. The indications of belantamab mafotin (BM), and tisotumab vedotin (TV) was relapsed/refinery multiple myoma, or current or metastatic cancer, respectively. The belantamab mafotin (BM) was by FDA in 2020 and EMA in 2020. The tisotumab vedotin (TV) was by FDA in 2021, but not approved by EMA (Sun et al., 2023).

**FIGURE 1:**
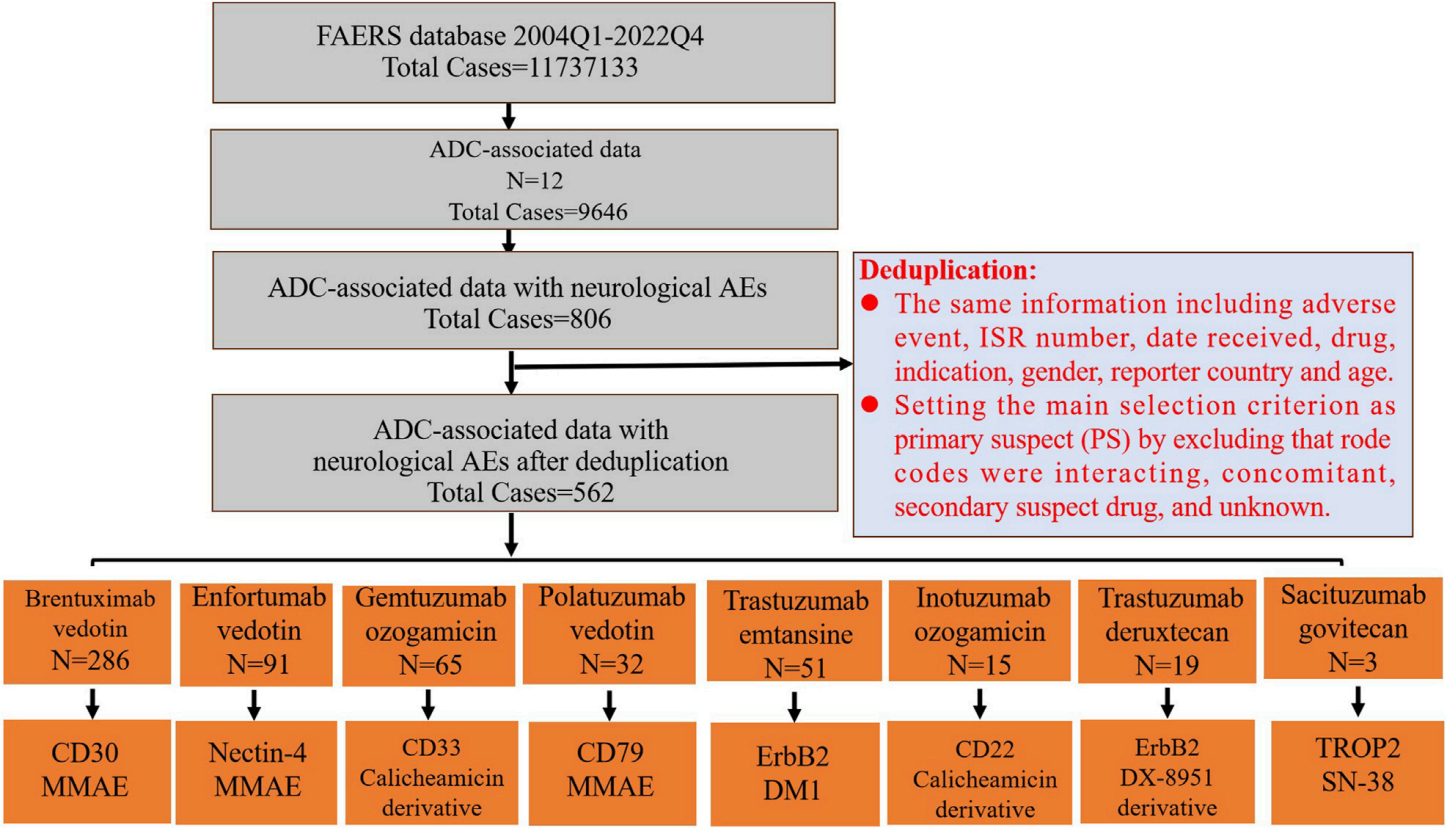
The FAERS database’s pipeline flowchart for screening ADC-associated neurological adverse events and the target antigen and payload of ADCs. The last row’s boxes include the target antigen in the first row and the payload in the second row.

The mechanism of ADCs involve two steps: first, the antibody binds to the target antigen on the cell surface through the antigen-binding fragment, causing the ADC to internalize with the aid of endocytosis; second, once inside the tumor cells, ADC partially releases the chemotherapeutic drugs through the cleavage and proteohydrolysis of lysosome ligands, which in turn breaks down microtubule proteins or DNA, effectively killing the tumor cells (Drago et al., 2021). There are differences in the targeted antigens and effective loads of ADC drugs (Figure 1). Monomethyl auristatin E (MMAE) is the payload of enfortumab vedotin, brentuximab vedotin, polatuzumab vedotin, and tisotumab vedotin. Derivative mertansine (DM1) is the payload of trastuzumab emtansine. The payloads of gemtuzumab ozogamicin, and inotuzumab ozogamicin are calicheamicin derivative. Pyrolobenzodiazepine (PBD) and CD-19 are the target antigen and payload of loncastuximab tesirine, respectively. Monomethyl auristatin F (MMAF) and BCMA are the target antigen and payload of belantamab mafodotin, respectively. The target antigen and payload of moxetumomab pasudotox are CD-22 and *Pseudomonas* exotoxin A (PE38). MMAE, DM1, and MMAF inhibit tubulin polymerization, whereas calicheamicin derivative and PBD cause DNA damage (Lee, 2021).

The toxicity of ADCs mainly includes blood toxicity, eye toxicity, peripheral neurotoxicity, skin toxicity, and gastrointestinal toxicity (Saber et al., 2019). Possible mechanisms include “off-target effects of non-tumour cells (off-target correlation),” “target antigen-specific uptake of non-tumour cells (target correlation),” or “non-target antigen uptake of non-tumour cells” (Mahalingaiah et al., 2019; Endo et al., 2021). The majority of ADC toxicity is thought to be derived from the payload (Donaghy, 2016). Studies have indicated that DM1 is linked to thrombocytopenia and hepatotoxicity, MMAF and DM4 to ocular toxicity, and MMAE to anemia, neutropenia, and peripheral neuropathy (Donaghy, 2016; Masters et al., 2018).

Central and peripheral neurotoxicities due to systemic antineoplastic therapy are common and often dose-limiting (Jordan et al., 2020). The neurotoxicity of ADCs, such as peripheral neuropathy, progressive multifocal leukoencephalopathy, intracranial haemorrhage, headache, and dizziness, has been reported (Takahashi et al., 2020; Coleman et al., 2021; FDALabel, 2022d; Cortes et al., 2022; FDALabel, 2023a; Song et al., 2023). In a phase III trial of brentuximab vedotin in Hodgkin’s lymphoma patients with a high risk of recurrence or progression after autologous hematopoietic stem cell transplantation, 67% of the patients experienced some level of peripheral neuropathy (Clifford et al., 2018). A phase II clinical trial of enfortumab vedotin for the treatment of urothelial carcinoma showed that 50% of the patients developed peripheral neuropathy (Rosenberg et al., 2019). Therefore, with the advent of ADCs, attention should be paid to any neurotoxicity caused by ADCs.

However, there is still a lack of comprehensive research on the neurological adverse events (AEs) associated with ADCs. The US FDA Adverse Event Reporting System (FAERS) serves as a repository for post-marketing adverse drug events and facilitates the FDA’s safety oversight of post-marketing drugs. Pharmacovigilance is a great way to discover associations between post-marketing drugs and adverse events. Mining based on the huge FAERS data allows for better discovery of real-world safety information. This study utilized FAERS real-world data to comprehensively analyze the neurological adverse events of post-marketing ADCs in order to investigate the relationship between ADCs and neurological adverse events as well as the factors affecting them, to compare the differences in neurological AEs between different ADCs, and to provide reference for clinical drug administration.

## 2 Materials and methods

### 2.1 Data sources and processing

The FAERS database is a freely accessible public database containing millions of adverse event reports from healthcare professionals, drug manufacturers, and others (Chen et al., 2023). Our pharmacovigilance study obtained ADC-associated neurological adverse events (AEs) from the first quarter of 2004 to the fourth quarter of 2022 using OpenVigil 2.1, a web-based query tool for physicians and pharmacists that provides intuitive access to the FAERS pharmacovigilance data (Bohm et al., 2021). OpenVigil 2.1 was used to query the FAERS database which uses cleaned data (removal of duplicates and missing information) (Sharma and Kumar, 2022).

In this study, we selected the following drugs for research based on the authorized time: brentuximab vedotin, enfortumab vedotin, gemtuzumab ozogamicin, polatuzumab vedotin, trastuzumab emtansine, inotuzumab ozogamicin, trastuzumab deruxtecan, sacituzumab govitecan, belantamab mafodotin, tisotumab vedotin, loncastuximab tesirine, and moxetumomab pasudotox. All the AEs were classified using the Medical Dictionary of Regulatory Activities (MedDRA; version 25.1), and the PTs were allocated according to systemic organ classes (SOCs). MedDRA has five levels from low to high: the lowest-level term (LLT), preferred term (PT), high-level term (HLT), high-level group term (HLGT), and system organ class (SOC). The PTs of all the neurological adverse events were acquired, with the SOC as “nervous system disorders.”

The comprehensive screening procedure is depicted in Figure 1. Duplicate reports were eliminated if they contained the same information, such as adverse events, ISR number, date received, medication, indication, sex, reporting nation, and age. After excluding potential neurological AEs that may occur due to concomitant medications and drug interactions, the remaining reports were further filtered by making the main selection criterion (ADCs) the primary suspect (PS). After the above deduplication process, the remaining reports were used for follow-up analysis.

### 2.2 Data mining

In this study, a disproportionality analysis was performed to evaluate the potential association between AEs and ADCs using the reporting odds ratio (ROR) and proportional reporting ratio (PRR) (Table 1).

**TABLE 1:** Two algorithms used for signal detection.

Algorithms	Equation	Criteria
ROR	ROR=(a/c)/(b/d)	a≥3, 95%CI ≥ 1
	95%CI = e^ln (ROR) ± 1.96 (1/a + 1/b + 1/c + 1/d)^0.5^	
PRR	PRR = [a/(c + d)]/[c/(a+b)]	a≥3, PRR≥2, χ^2^ ≥ 4
	χ^2^ = [(ad-bc)^2](a+b + c + d)/[(a+b) (c + d) (a+c) (b + d)]	

Equation: a, number of reports containing both the target drug and target adverse drug reaction-b, number of reports containing other adverse drug reaction of the target drug-c, number of reports containing the target adverse drug reaction of other drugs-d, number of reports containing other drugs and other adverse drug reactions. 95%CI, 95% confidence interval- χ^2^,chi-squared.

A disproportionality analysis can be used to evaluate possible associations between specific adverse events and specific drugs (Rothman et al., 2004; Ooba and Kubota, 2010; Caster et al., 2020). The disproportionality analysis was based on a comparison between the observed and expected number of adverse events for each drug and adverse event. For the ROR, a significant correlation is detected when the number of cases is ≥3 and the lower limit of the bilateral 95% confidence interval (95% CI) is >1. For the PRR, a significant correlation is detected when the number of cases is ≥3, the PRR is ≥2, and the chi-square is ≥4 (Tang et al., 2023). If both the ROR and PRR met the above criteria, the neurological adverse event signals were considered positive and associated with the corresponding drugs. We then collected data on the characteristics of the neurological adverse event cases related to ADC, including the number of annual reports, age, sex, reporting country, drug indications, and outcomes. We analyzed the signals of adverse neurological events related to ADCs and the mortality rate of the corresponding PT. Sensitivity analysis utilized raw data from individual case safety reports to eliminate the impact of concomitant medication use (Raghuvanshi et al., 2023).

### 2.3 Statistical analysis

All the data mining and statistical analyses were performed using Microsoft Excel 2019 and SPSS version 25.0. The chi-square test was used for intergroup comparisons. Serious outcomes included life-threatening events, hospitalization, disability, and death. Age, sex, and different treatment regimens were defined as exposure factors for both serious and non-serious neurological adverse events related to ADCs.

## 3 Results

### 3.1 Neurological adverse events among ADC users in FAERS from 2004 to 2022

**FIGURE 2:**
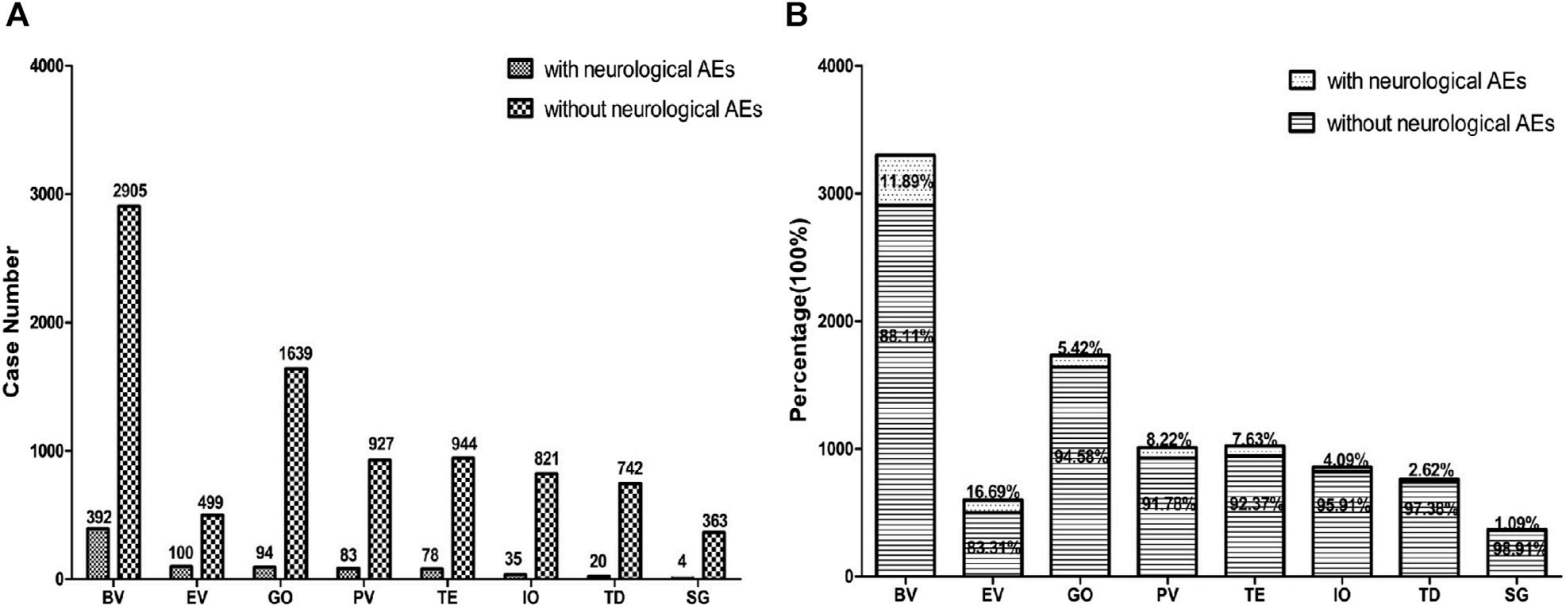
The bar chart above shows the number of reported neurological adverse events and without neurological adverse events of different ADCs in the FAERS database from 2004 to 2022 **(A)**. The proportional bar chart below shows the percentage of different ADCs neurological adverse events and without neurological adverse events in the FAERS database from 2004 to 2022 **(B)**. BV indicates brentuximab vedotin, EV indicates Enfortumab vedotin, GO indicates gemtuzumab ozogamicin, PV indicates polatuzumab vedotin, TE indicates trastuzumab emtansine, IO indicates inotuzumab ozogamicin, TD indicates trastuzumab deruxtecan, SG indicates sacituzumab govitecan.

We first extracted neurological AEs in patients receiving ADC treatment from the FAERS database from 2004 to 2022. The detailed data processing is shown in Figure 1. From the first quarter of 2004 to the fourth quarter of 2022, a total of 11737133 unique AEs were reported on FAERS, including 9,646 reports of ADCs (0.08% of all the reports). After excluding potential neurological AEs that may occur due to concomitant medications and drug interactions, we compiled reports of neurological AEs that considered ADCs as “primary suspicious drugs (PS)” and “secondary suspicious drugs (SS)” and obtained statistical data on the neurological AEs in patients treated with ADCs over the past 19 years. In reports related to ADCs, neurological AEs accounted for 8.36% (806/9,646) of the total AEs (Table 2). The incidence of neurological AEs varied among the ADCs. Enfortumab vedotin and brentuximab vedotin showed higher rates of adverse neurological events [16.69% (100/599) and 11.89% (392/3,297), respectively]. The incidence of neurological AEs for trastuzumab deruxtecan and sacituzumab govitecan was relatively low at 2.62% (20/762) and 1.09% (4/367), respectively (Figure 2). No neurological AEs associated with tisotumab vedotin, belantamab mafodotin, moxetumomab pasudotox, or loncastuximab tesirine were reported (see study limitations in *Discussion and Conclusion*).

**TABLE 2:** The counts of reports with ADCs related Neurological AEs yearly from 2004Q1 to 2022Q4.

Years	Neurological AEs	Non neurological AE	Total (PS + SS)
2004	7	101	108
2005	8	129	137
2006	11	215	226
2007	9	108	117
2008	11	75	86
2009	10	86	96
2010	6	93	99
2011	9	127	136
2012	10	145	155
2013	16	212	228
2014	38	305	343
2015	18	152	170
2016	31	219	250
2017	34	336	370
2018	46	549	595
2019	58	689	747
2020	87	820	907
2021	210	1785	1995
2022	187	2,234	2,421
Total	806	8,380	9,186

### 3.2 Descriptive analysis of cases of ADC-related neurological adverse events

We obtained 562 cases of neurological AEs with ADCs as the “primary suspect” from the FAERS database. The clinical characteristics of the patients are summarized in Table 3. The median patient age was 65 years old [(Min; Max) = 3; 92] from 295 available AE reports. Seven (1.25%), 140 (24.91%), and 148 (26.33%) patients were aged <18, 18–64, and >65 years, respectively (Table 3). In the ADC-related neurological AE reports, males accounted for 43.77% (246/562), females accounted for 35.41% (199/562), and 20.82% (117/562) of the cases had no sex information. In neurological AEs, the proportion of females with GO, TD, TE, and SG was 49.23% (32/65), 57.89% (11/19), 82.35% (42/51), and 100% (3/3), respectively. However, the proportion of females with IO, EV, BV, and PV was 26.67% (4/15), 19.78% (18/91), 26.91% (77/286), and 37.5% (12/32), respectively. The main reporting country was the United States (N = 264, 46.98%). A total of 19.22% (108/562) of the patients died. The indications for ADC-related neurological AEs were lymphoma (*n* = 232, 41.28%), leukaemia (*n* = 62, 11.03%), solid tumours (*n* = 120, 21.35%), other tumours (*n* = 14, 2.49%), and unknown tumours (*n* = 134, 23.84%).

**TABLE 3:** Characteristics of neurotoxicity correlated with ADCs.

Characteristics	Gemtuzumab ozogamicin (n, %)		Inotuzumab ozogamicin (n, %)	Enfortumab vedotin (n, %)	Trastuzumab deruxtecan (n, %)	Brentuximab vedotin (n, %)	Polatuzumab vedotin (n, %)	Trastuzumab emtansine (n, %)	Sacituzumab govitecan (n, %)	In total (n, %)
Gender										
Female	32 (49.23)		4 (26.67)	18 (19.78)	11 (57.89)	77 (26.92)	12 (37.50)	42 (82.35)	3 (100%)	199 (35.41)
Male	28 (43.08)		6 (40)	71 (78.02)	7 (36.84)	118 (41.26)	15 (46.88)	1 (1.96)	0	246 (43.77)
Unknown	5 (7.69)		5 (33.33)	2 (2.20)	1 (5.26)	91 (31.82)	5 (15.63)	8 (15.69)	0	117 (20.82)
Age										
Mean (SD)	56.15 (14.41)		44 (26.12)	74.80 (9.68)	67.66 (10.56)	54.5 (21.89)	73.65 (8.93)	60.28 (13.5)	57 (10)	59.65 (19.62)
Median [Min,Max]	57 [18–80]		56.5 [6–74]	76 [46–90]	69 [42–80]	60 [3–92]	73 [56–90]	57 [33–88]	57 [47–67]	65 [3–92]
Unknown	14		5	52	7	153	9	26	1	267
Age										
<18	0		2 (13.33)	0	0	5 (1.75)	0	0	0	7 (1.25)
18–64	34 (52.3)		5 (33.33)	6 (6.59)	4 (21.05)	71 (24.83)	3 (9.38)	16 (31.37)	1	140 (24.91)
>64	17 (26.15)		3 (20.00)	33 (36.26)	8 (42.11)	57 (19.93)	20 (62.50)	9 (17.65)	1	148 (26.33)
Unknown	14 (21.54)		5 (33.33)	52 (57.14)	7 (36.84)	153 (53.50)	9 (28.13)	26 (50.98)	1	267 (47.51)
Reporting country										
US	24 (36.92)		6 (40.00)	80 (87.90)	3 (15.79)	122 (42.66)	1 (3.13)	28 (54.91)	0	264 (46.98)
JP	5 (7.69)		3 (20.00)	11 (12.09)	14 (73.86))	63 (22.03)	19 (59.38)	5 (5.49)	0	120 (21.35)
FR	6 (9.23)		1 (6.67)	0	1 (5.26)	4 (1.40)	0	1 (1.96)	1	14 (2.49)
DE	3 (4.62)		1 (6.67)	0	0	6 (2.10)	0	1 (1.96)	1	12 (2.14)
United Kingdom	5 (7.69)		0	0	0	13 (4.55)	1 (3.13)	2 (3.92)	0	21 (3.74)
Others	22 (33.84)		4 (26.67)	0	1 (5.26)	78 (27.27)	11 (34.38)	14 (27.45)	1	141 (25.09)
Indications										
Leukaemia	51 (84.62)		11 (73.33)	0	0	0	0	0	0	62 (11.03)
Lymphoma	0		0	0	0	203 (70.98)	29 (90.63)	0	0	232 (41.28)
Solid tumor	0		0	55 (60.44)	17 (89.47)	0	0	45 (88.24)	3	120 (21.35)
Others	2 (3.08)		0	0	0	12 (4.20)	0	0	0	14 (2.49)
Unkown	12 (18.46)		4 (26.67)	36 (39.56)	2 (10.53)	71 (24.83)	3 (9.38)	6 (11.76)	0	134 (23.84)
Outcome										
Death	42 (64.62)		4 (26.67)	3 (3.30)	5 (26.32)	42 (14.69)	3 (9.38)	6 (11.76)	2 (66.67)	107 (19.22)
Hospitalization initial or prolonged	14 (21.54)		4 (26.67)	6 (6.59)	8 (42.11)	54 (18.88)	7 (21.88)	8 (15.69)	0	101 (17.97)
Life-threatening	6 (9.23)		0	0	0	10 (3.50)	3 (9.38)	0	0	19 (3.38)
Disability	0		1 (6.67)	1 (1.10)	0	15 (5.24)	1 (3.13)	1 (1.96)	0	19 (3.38)
Other	3 (4.62)		6 (40.00)	76 (83.52)	4 (21.05)	118 (41.26)	18 (56.250	22 (43.14)	1 (33.33)	246 (43.95)
Unkown	0		0	5 (5.49)	2 (10.53)	47 (16.43)	0	14 (27.45)	0	68 (12.10)

### 3.3 Scanning for ADC-related neurological adverse events

The number of neurological AEs associated with each specific ADC, as well as the corresponding ROR, PRR, and 95% CI are shown in Table 4. Compared to any other ADCs, brentuximab vedotin (N = 286, ROR = 5.648, 95% CI [4.992–6.389], PRR = 5.106, 95% CI [4.579–5.695], X2 = 961.13) and enfortumab vedotin (N = 91, ROR = 8.183, 95% CI [6.539–10.241), PRR = 7.03, 95% CI [5.824–8.487], X2 = 475.37) showed higher safety concerns regarding the nervous system, while sacituzumab govitecan (N = 3, ROR = 0.375, 95% CI [0.12–1.17], PRR = 0.381, 95% CI [0.123–1.175], X2 = 2.49) showed lower safety concerns regarding the nervous system. We screened for positive ADC-related neurological adverse event signals based on the above criteria for the ROR and PRR (Table 5). The neurological AE signals for brentuximab vedotin, gemtuzumab ozogamicin, anfortumab vedotin, polatuzumab vedotin, trastuzumab emtansine, trastuzumab deruxtecan, and inotuzumab ozogamicin were 11, 8, 5, 3, 3, 3, and 2, respectively.

**TABLE 4:** Safety adverse events among different ADC drugs.

Drug names	ADC-associated AEs n (%)	ADC-associated neurological AEs n (%)	ADC-associated neurological AEs as PSn (%)	ROR (95%CI)	PRR (95%CI)	X2
Brentuximab vedotin	3,297 (34.18)	392 (48.64)	286 (50.89)	5.65 (4.99–6.39)	5.11 (4.58–5.70)	961.13
Enfortumab vedotin	599 (6.21)	100 (12.41)	91 (16.19)	8.18 (6.54–10.24)	7.03 (5.82–8.49)	475.37
Gemtuzumabozogamicin	1733 (17.97)	94 (11.66)	65 (11.57)	2.52 (1.96–3.24)	2.44 (1.92–3.08)	54.79
Polatuzumab vedotin	1,010 (10.47)	83 (10.30)	32 (5.69)	3.00 (2.10–4.30)	2.87 (2.05–4.01)	38.05
Trastuzumab emtansine	1,022 (10.60)	78 (9.68)	51 (9.07)	3.27 (2.46–4.35)	3.11 (2.39–4.05)	72.60
Inotuzumab ozogamicin	856 (8.87)	35 (4.34)	15 (2.67)	1.45 (0.87–2.43)	1.44 (0.87–2.36)	1.62
Trastuzumab deruxtecan	762 (7.90)	20 (2.48)	19 (3.38)	1.13 (0.72–1.79)	1.13 (0.72–1.76)	0.17
Sacituzumab govitecan	367 (3.80)	4 (0.50)	3 (0.53)	0.38 (0.12–1.17)	0.38 (0.12–1.18)	2.49

**TABLE 5:** Signal strength of ADC-associated neurological AEs at the PT level in the FAERS database.

ADC	HLGT	Preferred term (PT)	Report number	ROR (95%CI)	PRR (95%CI)	X2
Brentuximab vedotin	peripheral neuropathies	neuropathy peripheral	137	15.92 (13.40–18.92)	15.09 (12.82–17.76)	1788.56
	peripheral neuropathies	peripheral sensory neuropathy	26	52.81 (35.80–77.92)	52.27 (35.57–76.79)	1,241.22
	peripheral neuropathies	polyneuropathy	25	26.16 (17.62–38.84)	25.90 (17.51–38.30)	570.48
	peripheral neuropathies	peripheral motor neuropathy	17	138.31 (85.17–224.60)	137.36 (84.86–222.33)	2097.47
	demyelinating disorders	progressive multifocal leukoencephalopathy	17	22.67 (14.05–36.58)	22.52 (14.01–36.21)	326.73
	peripheral neuropathies	guillain-barre syndrome	9	19.73 (10.24–38.03)	19.67 (10.23–37.81)	140.68
	encephalopathies	encephalopathy	9	3.85 (2.00–7.41)	3.84 (2.00–7.38)	16.18
	peripheral neuropathies	peripheral sensorimotor neuropathy	7	78.01 (36.89–164.94)	77.79 (36.87–164.13)	448.19
	peripheral neuropathies	peroneal nerve palsy	7	11.59 (5.51–24.35)	11.56 (5.51–24.24)	57.20
	neuromuscular disorders	autonomic neuropathy	4	43.59 (16.26–116.83)	43.52 (16.26–116.45)	125.05
	encephalopathies	leukoencephalopathy	3	6.82 (2.20–21.18)	6.81 (2.20–21.13)	9.62
Enfortumab vedotin	peripheral neuropathies	neuropathy peripheral	72	39.13 (30.55–50.11)	34.29 (27.62–42.56)	2,297.66
	neurological disorders	taste disorder	12	45.38 (25.60–80.45)	44.44 (25.37–77.84)	466.12
	neurological disorders	dysgeusia	5	2.72 (1.13–6.57)	2.71 (1.13–6.48)	5.40
	peripheral neuropathies	peripheral motor neuropathy	3	102.72 (32.93–320.49)	102.19 (32.95–316.91)	206.74
	peripheral neuropathies	peripheral sensory neuropathy	3	25.96 (8.34–80.80)	25.83 (8.35–79.91)	48.86
Gemtuzumab ozogamicin	central nervous system vascular disorders	cerebral haemorrhage	32	16.95 (11.93–24.09)	16.51 (11.73–23.25)	450.86
	neurological disorders	unresponsive to stimuli	6	4.56 (2.05–10.18)	4.54 (2.05–10.09)	13.23
	encephalopathies	encephalopathy	5	4.50 (1.87–10.82)	4.48 (1.87–10.75)	10.26
	central nervous system vascular disorders	haemorrhage intracranial	4	4.18 (1.56–11.15)	4.17 (1.57–11.09)	6.72
	central nervous system vascular disorders	subarachnoid haemorrhage	4	7.57 (2.83–20.20)	7.54 (2.83–20.07)	16.62
	central nervous system vascular disorders	cerebellar haemorrhage	3	37.89 (12.17–117.95)	37. 80 (12.18–117.31)	73.51
	peripheral neuropathies	guillain-barre syndrome	3	13.76 (4.43–42.76)	13.73 (4.43–42.53)	23.77
	central nervous system vascular disorders	haemorrhagic stroke	3	7.27 (2.34–22.58)	7.25 (2.34–22.46)	10.51
Polatuzumab vedotin	peripheral neuropathies	neuropathy peripheral	18	10.29 (6.42–16.47)	9.95 (6.32–15.65)	136.45
	encephalopathies	polyneuropathy	3	15.64 (5.03–48.69)	15.55 (5.03–48.08)	25.59
	central nervous system vascular disorders	cerebral haemorrhage	3	3.72 (1.20–11.57)	3.70 (1.20–11.44)	5.92
Trastuzumab emtansine	peripheral neuropathies	neuropathy peripheral	23	8.89 (5.87–13.47)	8.64 (5.78–12.92)	148.25
	neuromuscular disorders	muscular weakness	7	2.12 (1.01–4.46)	2.12 (1.01–4.46)	4.09
	encephalopathies	hepatic encephalopathy	3	10.33 (3.32–32.11)	10.29 (3.34–31.84)	16.72
Inotuzumab ozogamicin	encephalopathies	encephalopathy	6	13.93 (6.22–31.17)	13.76 (6.21–30.47)	58.81
	central nervous system vascular disorders	cerebral haemorrhage	3	3.97 (1.28–12.37)	3.95 (1.28–12.21)	4.00
Trastuzumab deruxtecan	central nervous system vascular disorders	cerebral haemorrhage	6	4.94 (2.21–11.02)	4.90 (2.21–10.88)	14.97
	neurological disorders	taste disorder	4	11.43 (4.28–30.56)	11.38 (4.28–30.25)	28.19

We classified the neurological AE signals according to the HLGT. The neurological AE signals of brentuximab vedotin include peripheral neuropathies (peripheral neuropathy, peripheral sensory neuropathy, polyneuropathy, peripheral motor neuropathy, Guillain-Barre syndrome, peripheral sensorimotor neuropathy, and peroneal nerve palsy), demyelinating disorders (progressive multifocal leukoencephalopathy), encephalopathies (encephalopathy and leukoencephalopathy), and neuromuscular disorders (autonomic neuropathy).

The neurological AE signals of enfortumab vedotin include peripheral neuropathies (peripheral neuropathy, peripheral motor neuropathy, and peripheral sensory neuropathy) and neurological disorders (taste and dysgeusia). Taste disorder is a new neurological AE signal of enfortumab vedotin that is not shown on the product label.

The neurological AE signals of gemtuzumab ozogamicin include central nervous system vascular disorders (cerebral haemorrhage, haemorrhage intracranial, subarachnoid haemorrhage, cerebellar haemorrhage, and hemorrhagic stroke), peripheral neuropathies (Guillain-Barre syndrome), encephalopathies (encephalopathy), and neurological disorders (unresponsive to stimuli). Guillain–Barré syndrome, encephalopathy, and unresponsiveness to stimuli are new neurological AEs signal associated with gemtuzumab ozogamicin.

The neurological AE signals of polatuzumab vedotin include peripheral neuropathies (peripheral neuropathy and polyneuropathy) and central nervous system vascular disorders (cerebral haemorrhage). Cerebral haemorrhage is a new neurological AE signal associated with polatuzumab vedotin. The neurological AE signals of trastuzumab emtansine include peripheral neuropathies (peripheral neuropathy), neuromuscular disorders (muscular weakness), and encephalopathies (hepatic encephalopathy). Muscular weakness is a new neurological AE signal associated with trastuzumab emtansine.

The neurological AE signals of inotuzumab ozogamicin include encephalopathies (encephalopathy) and central nervous system vascular disorders (cerebral haemorrhage). Encephalopathy is a novel neurological AE signal associated with inotuzumab ozogamicin.

The neurological AE signals of trastuzumab deruxtecan include central nervous system vascular disorders (cerebral haemorrhage) and neurological disorders (taste disorders). Cerebral haemorrhage is a new neurological AE signal associated with trastuzumab deruxtecan.

**FIGURE 3:**
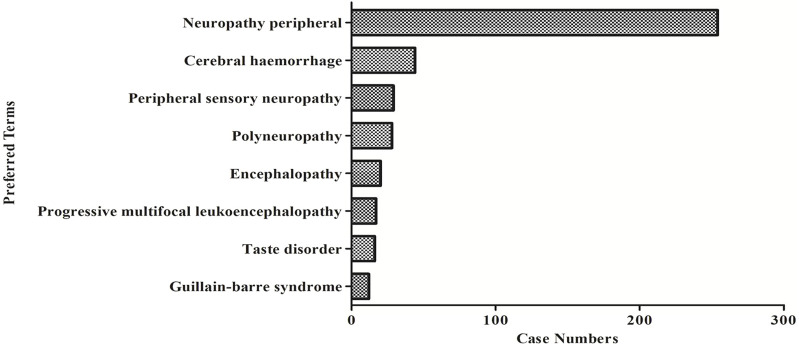
The number of reported cases of the first eight types of ADC related neurological AEs under different ADC treatment strategies.

**FIGURE 4:**
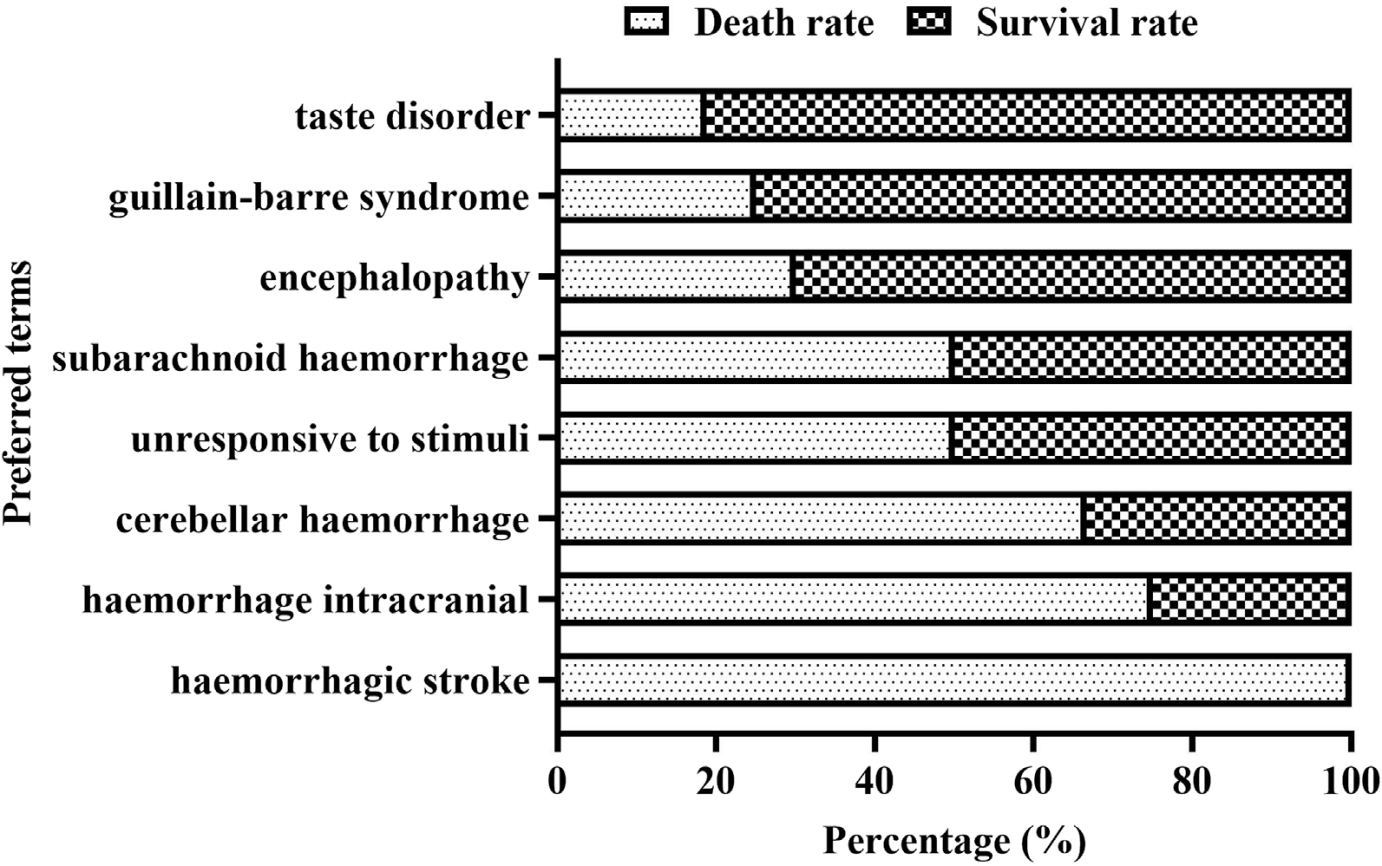
Death cases and their proportion in ADCs concomitantly with neurological AEs.

The eight most common ADC-related neurological AE signals are peripheral neuropathy [N = 250, ROR = 16.98, 95% CI (14.94–19.30), PRR = 16.0, 95% CI (14.21–18.09), X2 = 3,499.08], cerebral haemorrhage [N = 44, ROR = 9.45, 95% CI (7.01–12.73), PRR = 9.32, 95% CI (6.95–12.50), X2 = 318.02], peripheral sensory neuropathy [N = 29, ROR = 47.87, 95% CI (33.13–69.19), PRR = 47.43, 95% CI (32.93–68.30), X2 = 1,254.49], polyneuropathy [N = 28, ROR = 26.01, 95% CI (18.61–36.33), PRR = 25.75, 95% CI (18.50–35.86), X2 = 801.41], encephalopathy [N = 20, ROR = 5.16, 95% CI (3.32–8.01), PRR = 5.14, 95% CI (3.32–7.96), X2 = 62.55], progressive multifocal leukoencephalopathy [N = 17, ROR = 22.67, 95% CI (14.05–36.58), PRR = 22.52, 95% CI (14.01–36.21), X2 = 326.73], taste disorder [N = 16, ROR = 26.09, 95% CI (15.92–42.76), PRR = 25.78, 95% CI (15.83–42.00), X2 = 355.78], and guillain barrier syndrome [N = 12, ROR = 17.844, 95% CI (10.11–31.51), PRR = 17.79, 95% CI (10.09–31.35), X2 = 172.65] (Figure 3; Table 6). We also found that death is more common in some ADCs concomitantly with neurological AEs. The mortality rate of the ADCs concomitantly with nervous system signals was 100% for hemorrhagic stroke, 75% for internal haemorrhage, 66.67% for cerebellar haemorrhage, and 50% for unresponsive stimuli and subarachnoid haemorrhage (Figure 4). The results of sensitivity analysis are listed in Table 7. Find the accompanying drugs for each drug in OpenVigil2.1 and check their listing. After excluding cases accompanied by medication, the number of cases has decreased.

**TABLE 6:** The ROR and PRR values of the first eight types of ADC related neurological AEs under different ADC treatment strategies.

Preferred term (PT)	ROR (95%CI)	PRR (95%CI)	X2
neuropathy peripheral	16.98 (14.94–19.30)	16.04 (14.21–18.09)	3,499.08
cerebral haemorrhage	9.45 (7.01–12.73)	9.34 (6.95–12.49)	318.02
peripheral sensory neuropathy	47.87 (33.13–69.19)	47.43 (32.93–68.30)	1,254.49
polyneuropathy	26.01 (18.61–36.33)	25.75 (18.49–35.86)	801.41
encephalopathy	5.16 (3.32–8.01)	5.14 (3.32–7.96)	62.55
progressive multifocal leukoencephalopathy	22.67 (14.05–36.58)	22.52 (14.01–36.21)	326.73
taste disorder	26.09 (15.92–42.76)	25.78 (15.83–42. 00)	355.78
guillain-barre syndrome	17.84 (10.11–31.51)	17.79 (10.09–31.35)	172.65

**TABLE 7:** Sensitivity analysis after exclusion of cases of concomitant drugs.

Drug name	PT	Concomitant drugs	Cases with concomitant drugs	Cases without concomitant drugs	ROR (95%CI) without concomitant drugs
Gemtuzumab ozogamicin	cerebral haemorrhage	heparin	32	30	15.86 (11.03–22.80)
	cerebral haemorrhage	idarubicin	32	30	15.86 (11.03–22.80)
	cerebellar haemorrhage	heparin	3	2	25.20 (6.28–101.10)
	subarachnoid haemorrhage	idarubicin	4	3	5.67 (1.82–17.61)
	encephalopathy	acyclovir	5	4	3.59 (1.35–9.59)
Trastuzumab emtansine	neuropathy peripheral	ciclosporin	23	22	8.49 (5.55–12.98)
	neuropathy peripheral	vinorelbine	23	22	8.49 (5.55–12.98)
	neuropathy peripheral	capecitabine	23	22	8.49 (5.55–12.98)
	neuropathy peripheral	docetaxel	23	22	8.49 (5.55–12.98)
	neuropathy peripheral	paclitaxel	23	22	8.49 (5.55–12.98)
	muscular weakness	temazepam	7	6	1.81 (0.82–4.05)
Inotuzumab ozogamicin	encephalopathy	methotrexate	6	3	6.91 (2.22–21.52)
Enfortumab vedotin	neuropathy peripheral	carboplatin	72	71	38.50 (30.02–49.39)
	neuropathy peripheral	pregabalin	72	71	38.50 (30.02–49.39)
	neuropathy peripheral	cisplatin	72	70	37.88 (29.49–48.67)
Brentuximab vedotin	peripheral sensory neuropathy	ifosfamide	26	25	50.74 (34.13–75.42)
	polyneuropathy	cyclophosphamide	25	22	22.97 (15.08–34.99)
	neuropathy peripheral	cyclophosphamide	137	136	15.80 (13.29–18.79)
	neuropathy peripheral	itraconazole	137	136	15.80 (13.29–18.80)
	neuropathy peripheral	insulin aspart	137	136	15.80 (13.29–18.81)
	neuropathy peripheral	rosuvastatin	137	135	15.80 (13.29–18.82)
	neuropathy peripheral	amlodipine	137	136	15.80 (13.29–18.83)
	neuropathy peripheral	pentamidine	137	136	15.80 (13.29–18.84)
	neuropathy peripheral	carboplatin	137	136	15.80 (13.29–18.85)
	neuropathy peripheral	valganciclovir	137	136	15.80 (13.29–18.86)
polatuzumab vedotin	neuropathy peripheral	vincristine	18	1	9.70 (5.98–15.73)
	cerebral haemorrhage	clopidogrel	3	1	2.47 (0.62–9.92)

### 3.4 Comparison between serious and non-serious groups and risk factors for ADC-related neurological AEs

In cases of ADC-related neurological adverse events, over 43.77% of the patients had serious outcomes. Brentuximab vedotin (X2 = 121.5, *p* < 0.0001) and an indication of lymphoma (χ ^2^ = 75.42, *p* < 0.0001) were significantly more likely to occur in serious neurological AE cases (Table 8). The proportion of males and females with severe AEs was 44.72% (110/246) and 41.06% (101/246), respectively, with no statistical difference (χ ^2^ = 1.61, *p* = 0.2047); there was no difference in age between the two groups (χ ^2^ = 1.61, *p* = 0.7369).

**TABLE 8:** Differences in clinical characteristics between serious and non serious reports.

Clinical characteristics	Serious cases (N = 246)	Non-serious cases (N = 316)	Total (N = 562)	X2	*p*-value
Gender				1.61, 1	0.2047
Female	101 (41.06%)	98 (31.01%)	199 (35.41%)		
Male	110 (44.72%)	136 (43.03%)	246 (43.77%)		
Unknown	35 (14.23%)	82 (25.95%)	117 (20.82%)		
Age					
Mean (SD)	58.84 (19.68)	60.77 (19.64)	59.65 (19.62)		
Median [Min,Max]	64 [3–90]	66 [8–92]	65 [3–92]		
Unknown	75 (30.49)	192 (60.76)	267 (47.5)		
Age				1.33, 2	0.5150
<18	4 (1.63%)	3 (0.95%)	7 (1.25%)		
18–64	86 (34.96%)	54 (17.09%)	140 (24.91%)		
>64	81 (32.93%)	67 (21.20%)	148 (26.33%)		
Unknown	75 (30.49)	192 (60.75%)	267 (47.51%)		
Reporting country				55.57, 8	<0.0001
US	88 (35.77%)	181 (57.28%)	269 (47.86%)		
JP	71 (28.86%)	49 (15.51%)	120 (21.35%)		
FR	12 (4.88%)	3 (0.95%)	15 (2.67%)		
DE	8 (3.25%)	4 (1.27%)	12 (2.14%)		
BE	6 (2.44%)	0	6 (1.07%)		
IT	6 (2.44%)	4 (1.27%)	10 (1.78%)		
GB	5 (2.03%)	12 (3.79%)	17 (3.02%)		
AT	0	11 (3.48%)	11 (1.96%)		
Others	50 (20.33%)	5,216.46%)	102 (18.15%)		
Indications				75.42, 6	<0.0001
Leukaemia	54 (21.95%)	8 (2.53%)	62 (11.03%)		
Lymphoma	103 (41.86%)	126 (39.87%)	229 (40.75%)		
Breast cancer	21 (8.54%)	32 (10.13%)	53 (9.43%)		
gastric cancer	8 (3.25%)	0	8 (1.42%)		
bladder cancer	7 (2.85%)	44 (13.92%)	51 (9.07%)		
ureteric cancer	2 (0.81%)	0	2 (0.36%)		
Others	12 (4.88%)	10 (3.16%)	22 (3.91%)		
Unkown	39 (15.85%)	96 (30.38%)	135 (24.02%)		
Drugs				121.5 0	<0.0001
Brentuximab vedotin	121 (49.19%)	165 (52.22%)	286 (50.89%)		
Gemtuzumab ozogamicin	62 (25.20%)	3 (0.95%)	65 (11.57%)		
Trastuzumab deruxtecan	13 (5.28%)	6 (1.90%)	22 (3.38%)		
Polatuzumab vedotin	14 (5.69%)	18 (5.70%)	32 (5.69%)		
Trastuzumab emtansine	15 (6.10%)	36 (11.39%)	51 (9.07%)		
Enfortumab vedotin	10 (4.07)	81 (25.63%)	91 (16.19%)		
Inotuzumab ozogamicin	9 (3.66%)	6 (1.90%)	15 (2.67%)		
Sacituzumab govitecan	2 (0.81%)	1 (0.32%)	3 (0.53%)		

We further explored the risk factors that may affect the overall reporting of ADC-related neurological AEs using single-factor analysis (Table 9). The incidence of ADC-related neurological adverse events in males was higher than that in females, and the difference was statistically significant (χ 2 = 14.78, *p* = 0.0001). There was no difference in age between the two groups (χ ^2^ = 6.61, *p* = 0.0856). Brentuximab vedotin exhibited a significantly higher incidence of neurological adverse events (χ 2 = 171.00, P=<0.0001).

**TABLE 9:** Differences in clinical characteristics between neurological AEs and without neurological AEs reports.

Clinical characteristics	With neurological AEs (n, %)	Without neurological AEs (n, %)	X2	P
Gender				
Female	199 (35.41)	2,614 (41.01)	14.78, 1	0.0001
Male	246 (43.77)	2,207 (34.63)		
Unknown	117 (20.82)	1,553 (24.36)		
Age				
<18	7 (1.25)	107 (1.68)	4.24, 2	0.1202
18–64	140 (24.91)	1710 (26.83)		
>64	148 (26.33)	1,437 (22.54)		
Unknown	267 (47.51)	3,120 (48.95)		
Drugs			171.00, 7	<0.0001
Brentuximab vedotin	286 (50.89)	2,169 (34.03)		
Gemtuzumab ozogamicin	65 (11.57)	1,104 (17.32)		
Trastuzumab emtansine	51 (9.07)	667 (10.46)		
Polatuzumab vedotin	32 (5.69)	456 (7.15)		
Trastuzumab deruxtecan	19 (3.38)	718 (11.26)		
Enfortumab vedotin	91 (16.19)	476 (7.47)		
Inotuzumab ozogamicin	15 (2.67)	442 (6.93)		
Sacituzumab govitecan	3 (0.53)	342 (5.37)		
Total	562	6,374		

## 4 Discussion

Reports of ADC-related neurological adverse events are gradually increasing; however, comprehensive research is lacking. To the best of our knowledge, this is the first pharmacovigilance analysis of ADC-related neurological adverse events using FAERS data.

ADCs-associated neurological AEs were caused by the cytotoxic payloads, not the targeting antibody or linkers. In our study, the incidence of ADC-related neurological adverse events in males was higher than that in females. Neurological signals were detected in both microtubule polymerization inhibitors (brentuximab vedotin, enfortumab vedotin, polatuzumab vedotin, and trastuzumab emtansine) and DNA-damaging agents (gemtuzumab ozogamicin, inotuzumab ozogamicin, and trastuzumab deruxtecan). In addition, the data showed that compared to patients treated with other ADCs, those receiving microtubule polymerization inhibitors (brentuximab vedotin, enfortumab vedotin, polatuzumab vedotin, and trastuzumab emtansine) are more likely to develop neurotoxicity. We found that brentuximab vedotin- and gemtuzumab ozogamicin-related adverse neurological events were more likely to result in serious outcomes. No neurological AEs associated with tisotumab vedotin, belantamab mafodotin, moxetumomab pasudotox, or loncastuximab tesirine were reported. The above results are, to some extent, influenced by the time of drug launch. The reason for the zero neurological AE reports for belantamab mafodotin (approved in 2020), loncastuximab tesirine (approved in 2021), and tisotumab vedotin (approved in 2021) is mainly related to the short market time. The reason for the zero reported neurological adverse events (AEs) of moxizumab pasutuximab may be related to the lower number of patients receiving treatment after the drug was marketed.

Peripheral neuropathy is one of the most common adverse effects associated with ADCs. A meta-analysis showed that the incidence of peripheral neuropathy in ADCs is 39.6% (Zhu et al., 2023). In this study, peripheral neuropathy signals were detected using brentuximab vedotin, enfortumab vedotin, polatuzumab vedotin, gemtuzumab ozogamicin, and trastuzumab emtansine. Among these, brentuximab vedotin, enfortumab vedotin, and polatuzumab vedotin had the highest number of reports. The effective payloads of brentuximab vedotin, enfortumab vedotin, and polatuzumab vedotin is the tubulin inhibitor monolayer auristatin E (MMAE), trastuzumab emtansine is the tubulin inhibitor derivative mertansine (DM1), and gemtuzumab ozogamicin is a DNA damage calicheamicin derivative (Hafeez et al., 2020). Among all the ADCs, the G3/4 toxicity rate of peripheral neuropathy is relatively low but is most common in ADCs with an MMAE payload (6.5%) (Masters et al., 2018), which may lead to dose limitation or discontinuation (Saber and Leighton, 2015; Bae et al., 2021). Additionally, it has been shown to be unrelated to antibody targets (Saber and Leighton, 2015). Microtubules are important for maintaining highly elongated neuronal morphology and axonal transport, as well as the rapid movement of goods between neuronal cell bodies and distal nerve endings (Morfini et al., 2009). Peripheral neuropathy induced by MMAE ADCs is attributed to the nonspecific uptake of ADCs by peripheral nerves and the release of MMAE, which destroys microtubules (MT) and leads to neurodegeneration (Stagg et al., 2016). Cellular studies have shown that MMAE has a high affinity for the MT end, inducing structural defects, inhibiting MT kinetics, and reducing the degree of MT assembly while promoting the formation of microtubule protein loops. The inhibition of MT-dependent axonal transport mediated by MMAE ADCs leads to severe peripheral neuropathy (Best et al., 2021). The effective payloads of tisotumab vedotin (TV) is also MMAE (Markham, 2021). The effective payloads of Belantamab Mafodotin (BM) is the microtubule inhibitor monomethyl auristatin F (MMAF) (Markham, 2020). We were unable to obtain AE data for TV and BM due to the short launch period. However, the TV label specifies that peripheral neuropathy is a warning notice. 42% of people treated with TV develop peripheral neuropathy. For new or worsening peripheral neuropathy, the dose should be lowered or terminated following an examination (FDALabel, 2023b). However, the neurotoxicity of BM was not mentioned in the literature or instructions (Offidani et al., 2021). The effective payloads of loncastuximab tesirine is pyrrolobenzodiazepine, which is an alkylating agent. The effective payloads of moxetumomab pasudotox is *Pseudomonas* exotoxin A (PE38). The FDA labels and literature have not found any reports of loncastuximab tesirine (FDALabel, 2022a) or moxetumomab pasudotox (FDALabel, 2020a) causing peripheral neuropathy.

Central nervous AEs were primarily brain haemorrhages secondary to ADC-induced thrombocytopenia and various types of encephalopathies. We classified ADC-induced central nervous system haemorrhage (subarachnoid haemorrhage, intracranial haemorrhage, cerebral haemorrhage, and hemorrhagic stroke) and various encephalopathies (progressive multifocal leukoencephalopathy (PML), leukoencephalopathy, hepatic encephalopathy, and encephalopathy) as central nervous system toxicity. The data analysed in this study showed that the mortality rates of hemorrhagic stroke, internal haemorrhage, cerebral haemorrhage, and subarachnoid haemorrhage were relatively high and should be taken seriously. The following ADCs are ranked in descending order according to the number of hemorrhage PTs discovered: gemtuzumab ozogamicin, trastuzumab deruxtecan, inotuzumab ozogamicin, and polatuzumab vedotin. The following ADCs are ranked from high to low according to the number of encephalopathy PTs discovered: brentuximab vedotin, gemtuzumab ozogamicin, inotuzumab ozogamicin, and trastuzumab emtansine.

In the product label for gemtuzumab ozogamicin, haemorrhage is listed as a warning that may cause fatal haemorrhage. Gemtuzumab ozogamicin is a myelosuppressive drug that can cause fatal or life-threatening haemorrhages due to long-term thrombocytopenia (FDALabel, 2020b). In the ALFA-0701 (GO combined chemotherapy) trial, the incidence of haemorrhage in the GO group [118/131 (90.1%)] was significantly higher than that in the control group [107/131 (78.1%)] (*p* = 0.008). Grade ≥3 haemorrhage was reported by 30 patients (22.9%) in the GO group and 13 patients (9.5%) in the control group. Among the patients who died, the largest difference in hemorrhagic events was observed between these two groups [GO arm, 3 (2.3%); control arm, 1 (0.7%)] (Lambert et al., 2019). The main toxicity in the GO group was persistent thrombocytopenia with incidence rates of 16% and 3% in the GO and control groups, respectively (Castaigne et al., 2012). In a phase 3 INO-VAT study of inotuzumab ozogamicin, the incidence of thrombocytopenia at grade 3 and above was 41% (Kantarjian et al., 2019). In the INO trial, haemorrhage was observed as a complication of thrombocytopenia, with 33% of the patients experiencing hemorrhagic events. Five percent of the patients reported grade 3 or 4 haemorrhagic events (FDALabel, 2017) ^[28]^. However, there were no reports of cerebral haemorrhage. The risk of death from cerebral haemorrhage is high and should be seriously considered. The myelosuppression of trastuzumab deruxtecan is mainly manifested as neutropenia and anaemia, and the incidence of thrombocytopenia is lower (Li et al., 2022). In a study of transtuzumab deruxtecan (TD) for the treatment of HER2-positive breast cancer, the overall incidence of platelet count decline was 21.2%; the incidence of grade 3 was 3.8%, and grade 4 was 0.5% (Modi et al., 2020). There have been no reports of adverse reactions caused by TD in the literature. Our study is the first to uncover the presence of cerebral haemorrhage in patients with TD, suggesting that it may be caused by thrombocytopenia. No adverse hemorrhagic reactions were found in clinical trials of polatuzumab vedotin (PV) (Tilly et al., 2022), which may be related to strict subject selection. We found three cases of cerebral haemorrhage induced by PV in the FAERS database, and according to the ROR and PRR criteria, cerebral haemorrhage is a neurological signal related to PV. Both TD and PV were launched in the United States in 2019, and it is necessary to closely monitor platelet changes and haemorrhage adverse events when using these two drugs.

PML is a “Black Box” warning on the Brentuximab Vedotin (BV) label (FDALabel, 2023a). The three cases of PML caused by BV prompted manufacturers to include a black-box warning on the label (Wagner-Johnston et al., 2012). Subsequently, Carson et al. (Carson et al., 2014) reported five cases of PML caused by BV, all of which were associated with JC virus (John Cunningham polyoma virus) infection”, with a median onset time of 7 weeks following BV application. Our study identified 17 cases of PML associated with BV from the FAERS database from 2011 to 2022. Prior immunosuppressive therapy and a compromised immune system have been postulated to be risk factors. Potential mechanisms include a decrease in normal CD30-activated T cells and inhibition of the tumour necrosis factor (TNF) pathway. CD30 is a member of the TNF receptor, TNFα induces nuclear factors κ B (NF κ B) pathway. This pathway is involved in the transcription of the JC virus and has been identified in PML lesions. Blockade of CD30 may have downstream effects on TNFα, leading to viral activation (Wagner-Johnston et al., 2012). Our study also uncovered hepatic encephalopathy signals related to trastuzumab emtansine (TE). Hepatotoxicity is a “black box” warning on the TE instruction label (FDALabel, 2022b), and it may occur with a fatal risk in severe cases. Therefore, we focused on monitoring the liver function when applying TE. Our study found that GO and IO caused five and six cases of encephalopathy, respectively. However, encephalopathy was not recorded on the GO or IO labels. Marker et al. (Marker et al., 2020) reported a case of a multifocal necrotizing leukoencephalopathy variant, mainly characterized by superficial brainstem distribution and selective microglial cytotoxicity, associated with previous traditional chemotherapy treatment, namely CAR-T therapy, and inotuzumab ozogamicin. There have been no reports on the use of GO and IO associated encephalopathy. Therefore, considering the risk of encephalopathy, patients should pay attention to neurological symptoms when using GO and IO.

EV and TD have been implicated in the neurotoxicity of taste disorders. A Phase I clinical trial of EV for the treatment of urothelial carcinoma showed that dysgeusia is the most common treatment-related adverse event of EV (Takahashi et al., 2020). In a global, phase 2, single-arm clinical trial of EV for the treatment of urothelial carcinoma (Rosenberg et al., 2019), the incidence of dysgeusia was 40%, and there were no reports of ≥ grade 3 AEs. A meta-analysis (Guo et al., 2022) showed 9 cases of taste disorders in 8 clinical trials related to them. Taste disorder (ROR = 14.06) was a strong signal in the disproportionality analysis. Our research identified a new signal of muscle weakness in TE that requires more attention for clinical applications.

## 5 Conclusion

The FAERS data mining indicated an association between neurotoxicity and brentuximab vedotin, enfortumab vedotin, polatuzumab vedotin, trastuzumab emtansine, gemtuzumab ozogamicin, inotuzumab ozogamicin, and trastuzumab deruxtecan. This study has some limitations. First, the FAERS database is a self-reporting system that has some inherent selection biases; second, the market life of a drug has a big impact on the number of reporting cases; third, the disproportionality analysis based on the FAERS database only conducted a statistical evaluation of signal strength, but did not reveal whether there is a causal relationship between adverse signals and drugs. Further clinical studies are required to confirm these findings. With the widespread application of newly launched ADC drugs, combining FAERS data with other data sources is crucial for monitoring the sustained neurotoxicity of ADCs, including central and peripheral neurotoxicities. When administering ADC to patients with cancer, physicians should be aware of safety issues, such as dose adjustments due to peripheral neurotoxicity and death caused by central neurotoxicity, and should focus on early identification and prevention measures. Further studies are required to elucidate the mechanisms related to ADC neurotoxicity.

## Data Availability

The original contributions presented in the study are included in the article/Supplementary material, further inquiries can be directed to the corresponding authors.
